# Systematic Analysis of Evidence and Sound Expert Assessment: Two Enablers of Evidence‐Based Decision‐Making in Health

**DOI:** 10.1002/gch2.201800022

**Published:** 2018-06-28

**Authors:** Marie Paule Kieny, Vasee Moorthy

**Affiliations:** ^1^ Inserm Paris France; ^2^ Health Metrics and Measurement Cluster Department of Information Evidence and Research World Health Organization Geneva Switzerland

**Keywords:** advisory committees, evidence, policy, transparency

## Abstract

This commentary discusses attributes, membership, and modus operandi of advisory committees in the health sector, taking examples of a few committees operating internationally. It concludes on the importance of transparency and legitimacy for the credibility of their outcomes.

Three decades ago, expert opinion was accepted as good practice for the articulation of policies in health. Since then, there have been radical changes in the understanding of what should be considered as the norms to support health policy making. Indeed, scholars and policy‐makers alike have challenged the status quo, and the biased nature of expert opinion is now almost universally recognized. Although this article focuses on decision‐making in the health sector, avoidance of biases linked to expert opinion‐based processes has been a concern in other fields like education and the need for methodology improvement has been amply discussed.^1^ In the wake of this evolution, organizations that make policy recommendations in health at national or global level have adopted systematic reviews as the standard way of synthesizing evidence. This evidence is then usually assessed using the GRADE (Grading of Recommendations, Assessment, Development and Evaluation) methodology,^2^ which was primarily developed to determine the strength of evidence generated through randomized controlled trials (RCTs). When RCTs are not possible and for other types of designs used to analyze clinical observations, GRADE adaptations^3^ provide scientists with avenues to evaluate the strength of evidence.

But clearly, evidence itself is sometimes not sufficient to formulate health policies, and often scientific evidence remains difficult to understand for nonspecialists. It is accepted that values and priorities are important in setting goals for policy‐making. Moreover, it is rarely the case that the analysis of complex scientific problem resolves all uncertainties and generates evidence calling for unequivocal answers. Once the goals for policy‐making are agreed, a deliberative process is needed to identify the best options to achieve the goals. To facilitate this, evidence needs to be summarized and disseminated, and potential recommendations debated, crafted and made accessible to decision‐makers.^4^, ^5^ This step is most often performed by groups of individual experts generically called “advisory committee.” This raises the question: how can any review by a group of individuals avoid reintroducing the biases that systematic reviews and grading of evidence were intended to have eliminated in the process of evidence‐based decision making?

Advisory committees have very different ways of being constituted. Below are examples of advisory structures in three organizations operating on an international scale.

At the World Health Organization (WHO), selection of Expert Committees and Advisory committees or groups, whatever they are called, can follow very different procedures. Indeed, Expert committee members are drawn from Expert Panels to which only national governments can appoint members. It is nevertheless the prerogative of the WHO secretariat to decide which expert to draw from a Panel to form an Expert Committee, taking of course into consideration gender and geographical diversity. Appointment of WHO Advisory committees is often much less formal, and members can be picked up and chosen by the secretariat based on their individual expertise. Nevertheless, in some cases (as for the Strategic Advisory Committee on Vaccines and Immunization—SAGE), committee members are designated by a selection panel made of secretariat staff as well as partner organizations' representatives following an open, public call for nominations. This procedure is critical for transparency and is meant to also ensure representative legitimacy of selected committee members. This later point is of utmost importance—especially when advisory groups are concerned with decision‐making in areas with contested issues.

At the Wellcome Trust, Advisory committees are constituted by independent experts from around the world, and supplemented by members of the Board of Governors of the Trust and Wellcome Trust senior staff (not involved in activities considered by the Committee). Advisory committees can call on co‐opted expert members for specific advice where expertise is not already covered by the Committee.

At the European Commission, applications for membership of Horizon 2020 advisory groups are sought from national/international experts and the groups are constituted and appointed by the European Commission through review of individual competencies and expertise, ensuring balanced geographical representation and gender parity.

Like composition, advisory committees' modus operandi and acceptance of recommendations are very diverse. For example, WHO Expert Committees meet mostly in closed sessions. Although their recommendations are advisory in nature, the Director‐General needs to provide justifications to the Executive Board of the organization if she/he decides not to implement the advice received. Proceedings of meetings are formally published. Deliberations of the SAGE take place in sessions which are open, by invitation, to relevant stakeholders who are invited by the chair to express their views. Nevertheless, only members can participate in the crafting of recommendations. Like for Expert Committees, outcomes of SAGE meetings are published by WHO.

So, what does this all mean in term of avoidance of potential risk of bias? Real, potential and perceived conflicts of interests should be avoided^6^ in order to secure the legitimacy of the Advisory committee. Members should, as much as possible, be independent from the organization seeking advice. Whatever the mode of constitution, it is critical that the organization seeking advice and/or recommendations strives for a balance of technical expertise, experience, as well as opinions. It is therefore important that experts who could play the role of constructive critics or challengers of established “truths” (which can sometimes be incorrect) be considered as potential committee members. Also important is the need to avoid that outspoken committee members dominate the debates and ensure that silence is not accepted as a surrogate for agreement. Changing membership too often can be as detrimental as keeping asking the same experts over and over again. Indeed, committees need to operate in a trusted environment, which can take more than one meeting to be realized, but not in a context where all members are old friends, whose opinions are known by all participants even before they are verbalized. Often taken for granted, advisory committees should provide careful oversight of the risk of bias potentially introduced through the systematic review process. Indeed, systematic reviews are often done by junior research staff and only the consolidation is performed by the committee. The selection of which papers to include in the review is central and has to be overviewed in detail by the advisory committee. Finally, advisory committees need to consider—beyond the strength of evidence—whether their policy recommendations are suitable for the relevant audience, feasible, and acceptable in the specific context where implementation is envisaged. Affordability and other economic considerations are important and require specific additional contextually relevant processes to be taken into account in decision‐making.

To conclude, transparency on membership of advisory committees, on their mode of designation and operation, and on the processes used to generate recommendations is paramount in the credibility and generation of trust for the value of their outcomes. It is likewise important that any mode of designation considers members' representative legitimacy especially in areas with contested issues.

## Conflict of Interest

The authors declare no conflict of interest.
